# Healing beyond the body: Gestalt therapy as a catalyst for psychological resilience among breast cancer survivors

**DOI:** 10.6026/973206300213333

**Published:** 2025-09-30

**Authors:** Elilarasi Kamal, Kannan Jayaraman, Shankar Shanmugam Rajendran, Marudan Anbalagan, Jayalakshmi Lakshmanan, Gomathi Sivaprakash, Packiyalaksmi Muthuchamy

**Affiliations:** 1 Department of Psychiatric Nursing, College of Nursing, Madras Medical College, The Tamil Nadu Dr.MGR Medical University, Chennai, Tamil Nadu, India; 2 Department of Medical Oncology, Rajiv Gandhi Government General Hospital, The Tamil Nadu Dr.MGR Medical University, Chennai, Tamil Nadu, India; 3 Department of Paediatric Nursing, College of Nursing, Madras Medical College, The Tamil Nadu Dr.MGR Medical University, Chennai, Tamil Nadu, India

**Keywords:** Cancer, emotional well-being, gestalt therapy, self-perception, psychological resilience

## Abstract

Gestalt therapy was assessed for its impact on negative self-perception and eudaimonic well-being among breast cancer patients. A quasi-experimental study involving 60 participants revealed significant improvement in both self-perception and emotional well-being in the experimental group receiving Gestalt therapy, compared to the control group. The therapy's effectiveness was supported by the significant statistical differences in the outcomes (P < 0.05). Thus, Gestalt therapy is a promising non-pharmacological intervention to support psychological resilience and emotional healing in breast cancer patients, fostering self-acceptance and emotional growth.

## Background:

With breast cancer becoming increasingly prevalent worldwide, attention has shifted beyond its physical toll to the psychological struggles it brings [1]. Women facing this diagnosis often endure emotional distress, changes in body image and a shaken sense of identity. These challenges usually lead to negative self-perception, low self-worth, and emotional disconnection, all of which hinder recovery and overall well-being [2]. To provide complete cancer care, it is essential to address these psychological effects. At the same time, the concept of eudaimonic well-being, grounded in self-acceptance, autonomy, purpose in life and personal growth, has gained recognition as a crucial aspect of healing [3]. Supporting this kind of well-being in breast cancer patients plays a vital role in emotional recovery. However, this often requires focused psychological support tailored to the unique challenges of cancer [4]. Gestalt therapy, a client-centered and experiential approach, helps individuals become aware of their present emotions and resolve unresolved inner conflicts. Techniques like the empty chair method and role-play guide patients toward emotional clarity and self-integration [5]. Though Gestalt therapy has shown promise in other chronic illness settings, its role in oncology-particularly for improving self-image and deeper well-being-is still underused [6]. Therefore, it is of interest to explore the potential of Gestalt therapy to address the psychological challenges faced by breast cancer patients, particularly in terms of reducing negative self-perception and fostering eudaimonic well-being. By focusing on emotional and psychological support, this research aims to contribute to a more holistic approach to cancer care, emphasizing the need for interventions that promote self-acceptance, autonomy, and personal growth.

## Methodology:

A quasi-experimental design to assess the impact of Gestalt therapy on negative self-perception and eudaimonic well-being among women diagnosed with breast cancer. The research was carried out in the Oncology Unit of Rajiv Gandhi Government General Hospital (RGGGH), Chennai, after obtaining ethical clearance from the Institutional Ethics Committee of Madras Medical College and formal approval from the Dean. A total of 60 participants were selected using non-randomised convenience sampling. They were divided into two equal groups: the experimental group received Gestalt therapy, and the control group continued with routine standard care. Women who had been diagnosed with breast cancer, were willing to participate, and were able to attend the therapy sessions were included in the study. Those with severe cognitive impairments or psychiatric illness, or who were physically unfit to take part, were excluded. Gestalt therapy was delivered over two weeks, with daily 30-minute sessions involving techniques such as the empty chair method, body awareness practices, and role-playing exercises. These sessions were designed to help participants explore unresolved emotions, rebuild self-worth, and promote psychological healing. Data were collected at two points-before and after the intervention-using the Rosenberg Self-Esteem Scale to evaluate self-perception and Ryff's Psychological Well-Being Scale to assess emotional well-being. Background details like age, education level, comorbid health conditions, and type of cancer treatment were also gathered. Data analysis was performed using IBM SPSS software. Paired and independent t-tests were used to compare numerical scores within and between groups, while the Chi-square test was applied for categorical variables. A significance level of p ≤ 0.05 was used to interpret the findings.

## Results:

Participants in the study were mostly women between the ages of 41 and 50, with a majority having completed secondary education. Most were undergoing chemotherapy or combined treatments. Both experimental and control groups were similar in their demographic characteristics, with no significant differences at baseline. Before the intervention, both groups showed comparable levels of negative self-perception. After Gestalt therapy, 76.67% of participants in the experimental group showed high self-perception scores, indicating a positive change. In contrast, the control group showed no such improvement-46.67% had low scores, and 53.33% had moderate scores. The difference between the groups was statistically significant (P < 0.05), based on Chi-square test results. A notable pre- and post-test improvement was seen in the experimental group, while the control group showed no meaningful change. Pre-test results showed moderate levels of well-being in both groups with no significant difference. After the intervention, 80.00% of participants in the experimental group scored in the good category, while 20.00% had moderate scores. The control group did not show any improvement-43.33% had poor scores and 56.67% had moderate scores. This post-test difference was also statistically significant (P < 0.05). A negative correlation (r = -0.38, P = 0.001) was found between negative self-perception and eudaimonic well-being, indicating that improved self-perception was linked to better emotional well-being. Better outcomes were observed among participants aged 41-50, those with higher education, no comorbidities, and a family history of cancer. While these trends were clear in the experimental group, they were not statistically significant in the control group.

## Discussion:

The present study found that Gestalt therapy significantly improved negative self-perception and enhanced eudaimonic well-being among breast cancer patients, highlighting its potential as a valuable psychosocial intervention in oncology care. These results align with previous research, such as Rajendran *et al.* (2021) [7], who examined the psychosocial needs of breast cancer survivors in Chennai. Rajendran's study emphasized the challenges of emotional isolation, low self-esteem and ineffective stress coping, underscoring the necessity of psychological support to facilitate holistic recovery. The negative correlation between negative self-perception and eudaimonic well-being (r = -0.38, P = 0.001) observed in the current study further reinforces the connection between improved self-image and emotional resilience. Additionally, the findings are consistent with studies by Vala *et al.* (2022) [8], both of which demonstrated the effectiveness of Gestalt therapy in enhancing emotional clarity, hope and mental resilience in cancer patients. Nazari *et al.* [9] specifically noted the positive outcomes of Gestalt therapy in helping patients develop a stronger sense of self and emotional well-being. These findings collectively support the integration of Gestalt-based psycho-oncology care into clinical practice, particularly as a non-pharmacological strategy to foster emotional healing, self-acceptance and inner growth among women battling breast cancer.

## Conclusion:

Gestalt Therapy significantly improved negative self-perception and eudaimonic well-being among breast cancer patients. Participants who received structured therapy demonstrated meaningful psychological gains compared to those with routine care alone. These outcomes validate the therapy's strength in promoting self-awareness, acceptance and emotional healing. Gestalt Therapy offers a holistic, patient-centred model that should be integrated into psycho-oncology care to enhance both mental health and overall quality of life.
